# Costunolide alleviates hyperglycaemia‐induced diabetic cardiomyopathy via inhibiting inflammatory responses and oxidative stress

**DOI:** 10.1111/jcmm.17686

**Published:** 2023-02-21

**Authors:** Bo Jin, Yi Chen, Jiong Wang, Yue Chen, Mengpei Zhang, Jianxiong Huang, Yi Wang

**Affiliations:** ^1^ The Affiliated Xiangshan Hospital of Wenzhou Medical University Ningbo China; ^2^ Chemical Biology Research Center, School of Pharmaceutical Sciences Wenzhou Medical University Wenzhou China

**Keywords:** Costunolide, diabetic cardiomyopathy, inflammation, NF‐κB, Nrf‐2, oxidative stress

## Abstract

Hyperglycaemia‐induced myocardial injury promotes the induction of heart failure in diabetic patients. Impaired antioxidant capability and sustained chronic inflammation play a vital role in the progression of diabetic cardiomyopathy (DCM). Costunolide (Cos), a natural compound with anti‐inflammatory and antioxidant properties, has exhibited therapeutic effects in various inflammatory diseases. However, the role of Cos in diabetes‐induced myocardial injury remains poorly understood. In this study, we investigated the effect of Cos on DCM and explored the potential mechanisms. C57BL/6 mice were administered intraperitoneal streptozotocin for DCM induction. Cos‐mediated anti‐inflammatory and antioxidation activities were examined in heart tissues of diabetic mice and high glucose (HG)‐stimulated cardiomyocytes. Cos markedly inhibited HG‐induced fibrotic responses in diabetic mice and H9c2 cells, respectively. The cardioprotective effects of Cos could be correlated to the reduced expression of inflammatory cytokines and decreased oxidative stress. Further investigations demonstrated Cos reversed diabetes‐induced nuclear factor‐κB (NF‐κB) activation and alleviated impaired antioxidant defence system, principally via activation of nuclear factor‐erythroid 2 p45‐related factor‐2 (Nrf‐2). Cos alleviated cardiac damage and improved cardiac function in diabetic mice by inhibiting NF‐κB‐mediated inflammatory responses and activating the Nrf‐2‐mediated antioxidant effects. Therefore, Cos could be a potential candidate for the treatment of DCM.

## 1 INTRODUCTION

Diabetes mellitus (DM) is a common metabolic disorder worldwide and has gradually become an epidemic in recent years. Elevated blood glucose levels typically cause a series of tissue injury‐related events during diabetes, resulting in diabetic complications such as diabetic cardiomyopathy (DCM), diabetic nephropathy, diabetic foot disease and retinopathy. Among these diabetic complications, DCM is considered a risk factor of heart failure, known to be associated with disability and mortality in patients with diabetes.^1^ DCM persistently presents as diastolic dysfunction during the early stage, progressing to systolic dysfunction in the advanced phase. Hyperglycaemia‐induced cardiac fibrosis and hypertrophy are the most frequently proposed pathological mechanisms underlying structural and functional alterations in DCM.^2^

Subcellular and low‐grade inflammation caused by metabolic disturbances are key pathogenic features of diabetes.^3^ Studies have suggested that hyperglycaemia‐induced cardiac adverse remodelling is highly associated with hyperglycaemia‐induced inflammatory responses and oxidative stress.^4^ On one hand, hyperglycaemia activates nuclear factor‐κB (NF‐κB) and mitogen‐activated protein kinases (MAPKs), which are crucial regulators of pro‐inflammatory signalling pathways, resulting in the release of chemokines and inflammatory cytokines, such as monocyte chemoattractant protein (MCP)‐1, tumour necrosis factor (TNF)‐α, interleukin (IL)‐6 and IL‐1β, in the hearts of diabetic mice. Subsequently, the released cytokines recruit macrophages, which reside in the cardiac tissues. Infiltrated macrophages express additional inflammatory cytokines under hyperglycaemic conditions, which further aggravate myocardial inflammation and cardiac injury, contributing to the deterioration of DCM. On the contrary, numerous studies have shown that reactive oxygen species (ROS) levels are elevated in the diabetic hearts, which ultimately cause cardiac oxidative injury.^5^ Nuclear factor‐erythroid 2 p45‐related factor‐2 (Nrf‐2), a key transcription factor regulating antioxidant stress, plays an important role in the antioxidant response induction. Nrf‐2 is reportedly downregulated in the diabetic heart. Excessive ROS production and weakened antioxidant capacity cause mitochondrial damage and lipid peroxidation, contributing to cardiomyocyte damage in DCM.^6^ Thus, hyperglycaemia‐induced inflammation and oxidative stress are primary factors inducing myocardial cell death, thereby resulting in a range of downstream events associated with cardiac injury and remodelling. Thus, blocking inflammatory and oxidative responses may afford a potential strategy to hinder the development of DCM.

Costunolide (Cos), a natural sesquiterpene lactone, has been shown to possess diverse biological activities.^7^ Cos alleviates dextran sulfate sodium‐induced acute ulcerative colitis by inhibiting the NF‐κB signalling pathway.^8^ In addition, Cos attenuates heat‐killed *Staphylococcus aureus*‐induced acute lung injury via inhibition of macrophage activation and displays anti‐fibrotic activity in bleomycin‐induced pulmonary fibrosis by regulating NF‐κB and Nrf‐2/NADPH oxidase 4 (NOX4) signalling pathways.^9^, ^10^ These findings suggest that Cos could be a promising candidate for the treatment of DCM.

Herein, using a streptozotocin (STZ)‐induced diabetic mouse model and HG‐challenged H9c2 cardiomyocytes, we examined the potential cardioprotective effect of Cos against DCM and elucidated the underlying mechanisms.

## 2 MATERIALS AND METHODS

### 2.1 Reagents

**FIGURE 1:**
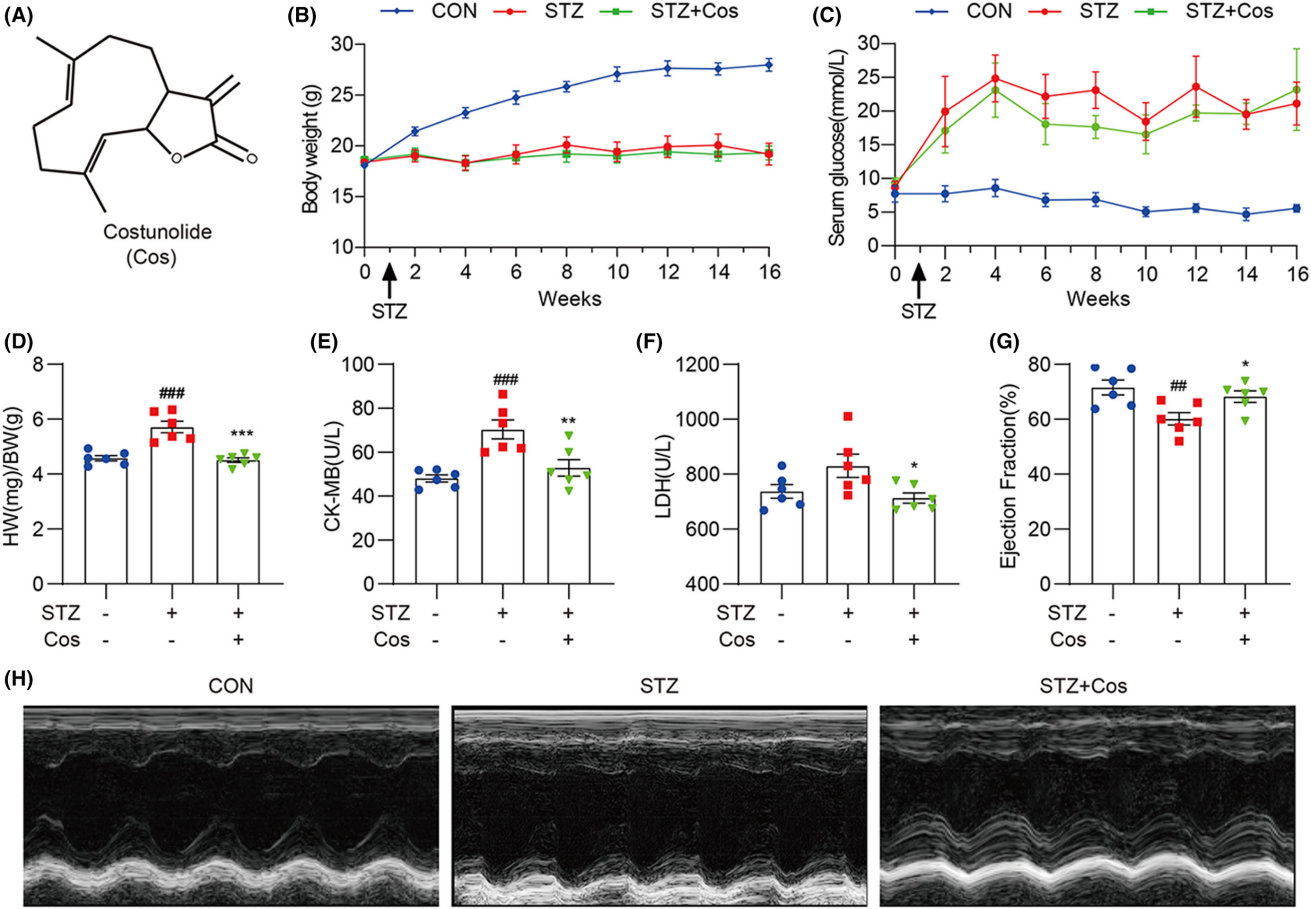
Cos improves cardiac function and alleviates hyperglycaemia‐induced cardiac pathological injuries. (A) Chemical structure of Cos. (B,C) Body weight (B) and serum glucose levels (C) of control and diabetic mice. (D) Heart weight/body weight ratio was established after sacrificing mice. (E,F) Serum creatine kinase (CK)‐MB (CK‐MB) and lactate dehydrogenase (LDH) levels were determined using biochemical detection kits. (G) Echocardiography showing the ejection fraction (%) of mice. (H) Representative images of M‐mode echocardiogram. Data are expressed as mean ± SEM, *n* = 6. ##*p* < 0.01, ###*p* < 0.001 between CON and STZ groups; *p < 0.05, **p < 0.01, ***p < 0.001 between STZ and STZ + Cos groups.

Costunolide was obtained from Chengdu Alfa Biotechnology Co.,Ltd (Chengdu, China) and dissolved in DMSO for experiments in vitro and in 0.5% CMC‐Na for studies in vivo. The chemical structure of Costunolide is shown in (Figure 1A). Antibodies against inhibitor of IκB‐α (1:1000, cat. no. 4814), NF‐κB P65 subunit (1:1000, cat. no. 8242), p‐P65 (1:1000, cat. no. 3033), p‐P38 (1:1000, cat. no. 9211), P38 (1:1000, cat. no. 9212) and GAPDH (1:1000, cat. no. 97166) were purchased from Cell Signaling Technology (Danvers, MA, USA). Anti‐TGF‐β (1:1000, cat. no. ab92486) and anti‐β‐MyHC (1:1000, cat. no. ab50967) antibodies were acquired from Abcam (Cambridge, MA, USA). Antibodies against MCP‐1 (1:200, cat. no. sc‐32771) and F4/80 (1:200, cat. no. sc‐377009) were obtained from Santa Cruz (CA, USA), and HO‐1 (1:1000, cat. no. 66743‐1‐Ig), COL‐1 (1:1000, cat. no. 66761‐1‐Ig) and Nrf‐2 (1:1000, cat. no. 16396‐1‐AP) were also purchased from Proteintech (Wuhan, China). The assay kits for CK‐MB (cat. no. E006‐1‐1), LDH (cat. no. A020‐2‐2) were obtained from Nanjing Jiancheng Bioengineering Institute (Nanjing, China). Biochemical detection kits of superoxide dismutase (SOD, cat. no. S0109) and malondialdehyde (MDA, cat. no. S01315) were acquire form Beyotime (Shanghai, China). ML385 was purchased from TargetMol Chemicals Inc. (Boston, MA, USA). Bovine serum albumin (BSA, cat. no. A1933) was purchased from Sigma (St. Louis, MO, USA). High glucose solution (1 M) was prepared. Dissolving 198.17 μg D‐ (+)‐glucose monohydrate (cat. no. 5250278, aladdin, USA) in 1 mL ddH_2_O, and then filtered with a 0.22 μm sieve.

### 2.2 Cell culture

The embryonic rat heart‐derived cell line, H9c2, was purchased from the Shanghai Institute of Biochemistry and Cell Biology (CSTR:19,375.09.3101RATGNR5, Shanghai, China). H9c2 cells were grown in Dulbecco's Modified Eagle Medium (cat. no. 11995040, Gibco; Eggenstein, Germany), containing 1 g/L glucose and supplemented with 12% foetal bovine serum (FBS; cat. no. 14160063, Gibco; Eggenstein, Germany), 100 U/mL penicillin and 100 mg/mL streptomycin (Invitrogen; CA, USA). The cells were incubated at 37°C in a 5% CO_2_ humidified incubator.

### 2.3 Animals

Male C57BL/6 mice (18–20 g) were obtained from the Animal Center of Wenzhou Medical University. All animal care and experimental procedures were approved by the Wenzhou Medical University Animal Policy and Welfare Committee (Approval Document No. wydw2019‐0145). Mice were maintained at 22°C and 60% relative humidity and fed a standard rodent diet and purified water.

Mice were subsequently divided into three groups (*n* = 6 per group): non‐diabetic control group (CON), STZ‐induced diabetic group (STZ) and Cos‐treated STZ‐induced diabetic group (STZ + Cos). After a one‐week adaptation, diabetes was induced in the STZ and STZ + Cos groups by administering a single intraperitoneal injection of STZ (cat. no. B2001, Boaigang Biological Technology Co., Ltd. Beijing, China) at the dose of 170 mg/kg dissolved in 0.5% citrate buffer (pH 4.5). The control mice received an intraperitoneal injection of equivalent citrate buffer. Tail blood glucose levels were measured once every 2 weeks after a 12 h fasting period. Mice with fasting‐blood glucose ˃12.2 mmol/L for 8 weeks were considered hyperglycaemic, indicating the successful establishment of the diabetic model. The mice from STZ + Cos group were administered 20 mg/kg Cos orally, once every 2 days. Eight weeks after Cos treatment, mice were sacrificed, and heart tissues and serum were harvested.

### 2.4 Echocardiographic measurements

One week prior to sacrifice, echocardiography was performed in M‐mode using Vevo 770™ High‐Resolution Imaging System (Visualsonics, Toronto, Canada). Left ventricular ejection fraction and left ventricular fractional shortening were expressed as percentages (Vevo 2100, Visual Sonics imaging system, Toronto, ON, Canada). LV end‐diastolic volume (LVVd), ejection fraction (EF) and LV Mass (AW) were automatically calculated by the ultrasound machine.

### 2.5 MTT assay

MTT powder (cat. no. M8180, Solarbio; Beijing, China) was dissolved in phosphate‐buffered saline. H9c2 cells (1 × 10^4^ per well) were seeded in 96‐well plates. After cell adherence, Cos was added to the wells at various doses (2.5–20 μM), followed by incubation for 48 h. Next, MTT was added to each well (1 mg/mL) and incubated for 4 h. The formazan crystals were then dissolved in dimethyl sulfoxide (DMSO; 150 μL/well), and absorbance was examined at 490 nm using SpectraMax M5 microplate reader (Molecular Devices, CA, USA).

### 2.6 Histological and immunohistochemical analyses

Harvested heart tissues were fixed in 4% buffered formalin, subsequently embedded and sliced into 5 μm thick sections for histologic analyses. Haematoxylin–eosin (cat. no. G1120), Sirius red (cat. no. S8060) and Masson's trichrome (cat. no. G1340) staining were performed according to the respective kit instructions (Solarbio).

For immunohistochemistry, antigen retrieval was performed after deparaffinization and hydration of sections. Next, sections were blocked with 5% bovine serum albumin for 1 h, followed by overnight incubation with F4/80 antibody (1:50) at 4°C. After overnight incubation, sections were incubated with horseradish peroxidase (HRP)‐conjugated secondary antibodies (cat. no. A0216, Beyotime). Finally, tissues were counterstained with haematoxylin and covered with neutral resin. Images were captured using a Nikon microscope (Nikon, Tokyo, Japan), and the mean percentage of the staining‐positive area was measured and calculated using the ImageJ software (National Institutes of Health, Bethesda, MD).

### 2.7 Immunofluorescence

H9c2 cells were pretreated with 2.5 or 5 μM Cos for 1 h, followed by exposure to 33 mM glucose for 3 h. Cells were fixed with 4% paraformaldehyde for 15 min and permeabilized with 0.5% Triton X‐100 for 10 min. Subsequently, cells were incubated with an anti‐p65 antibody (1:1000) at 4°C overnight. A FITC‐conjugated secondary antibody was used for detection. Finally, nuclei were counterstained with an anti‐fluorescent quencher containing DAPI. Images were observed and captured using an inverted fluorescence microscope (TE2000U, Nikon, Tokyo, Japan).

### 2.8 Rhodamine‐phalloidin staining

H9c2 cells were pretreated with 2.5 or 5 μM Cos for 1 h, followed by exposure to 33 mM glucose for 36 h. Cells were fixed with 4% paraformaldehyde and permeabilized with 0.5% Triton X‐100 for 10 min. Subsequently, cells were stained with rhodamine‐phalloidin (cat. no. CA1610, Solarbio) for 30 min. Finally, nuclei were counterstained with an anti‐fluorescent quencher containing DAPI. Images were observed and captured using a fluorescence microscope (Nikon).

### 2.9 Determination of ROS generation

Dihydroethidium staining (DHE, Beyotime) was utilized to detect ROS generation as previously described.^11^ Heart tissue sections were incubated with 2 μM DHE at 37°C for 1 h in a dark humidified chamber. For the in vitro experiment, H9c2 cells were incubated with 2 μM DHE at 37°C for 30 min in a dark humidified chamber, Fluorescent images were obtained using fluorescence microscope (Nikon).

### 2.10 Measurement of enzyme activities

Serum CK‐MB, LDH and myocyte enzyme activities of SOD and MDA were measured using corresponding detection kits according to the manufacturers' instructions.

### 2.11 Reverse transcription‐quantitative PCR (RT‐qPCR)

Cultured cells or heart tissue samples were lysed with TRIzol (cat. no. 15596026, Thermo Fisher; CA, USA). Total RNA was extracted and separated using chloroform and isopropanol and purified with ethanol. Reverse transcription was performed using the PrimeScript™ RT reagent Kit (cat. no. DRR037A, Takara Bio Inc., Kusatsu, Japan). Quantitative PCR was performed using TB Green® Premix Ex Taq™ II (cat. no. RR820B, Takara Bio Inc.). Primers (Table S1) were obtained from Sangon Biotech (Shanghai, China). mRNA levels were detected and normalized using β‐actin as the loading control.

### 2.12 Western blot analysis

Cells or heart tissue samples were lysed using RIPA buffer. Total proteins were separated using 10% sodium dodecyl sulfate‐polyacrylamide gel electrophoresis (SDS‐PAGE) and then electro‐transferred to polyvinylidene fluoride (PVDF) membranes (cat. no. 1620177, Bio‐Rad Laboratory; Hercules, CA). Membranes were blocked in 5% non‐fat milk for 2 h at room temperature and cut into bands, followed by incubation with respective antibodies. After overnight incubation, protein bands were incubated with HRP‐conjugated secondary antibodies and scanned using an image analyser (Quantity One System; Bio‐Rad, Richmond, CA, USA).

### 2.13 Statistical analysis

All data were presented as mean ± SEM. Prism 8.0 software (GraphPad, San Diego, CA, USA) was used for the statistical analysis. Student's *t*‐test was used to compare two groups of data. One‐way anova followed by Dunnett's *post hoc* test was used to compare more than two groups of data. A *p* value < 0.05 was considered as significant.

## 3 RESULTS

### 3.1 Cos improves cardiac function and alleviates hyperglycaemia‐induced cardiac pathological injuries

To explore the therapeutic effect of Cos on DCM‐mediated cardiac injury, we established a type 1 diabetic mouse model by administering STZ intraperitoneally. Compared with the diabetic group, Cos treatment at the dose of 20 mg/kg did not alter body weight and serum glucose levels in STZ + Cos group mice (Figure 1B,C). After persistent hyperglycaemia for 16 weeks, diabetic mice showed an increased ratio of heart weight to body weight when compared to those of non‐diabetic control mice (Figure 1D). However, Cos treatment significantly reduced this ratio in diabetic mice, indicating that Cos could reverse diabetes‐induced cardiac hypertrophy. Serum levels of lactate dehydrogenase (LDH) and creatine kinase (CK)‐MB are common indicators of heart damage.^12^ We found that hyperglycaemia increased serum LDH and CK‐MB levels, which were reversed by Cos treatment (Figure 1 E,F). In addition, Cos reduced HG‐induced upregulation of brain natriuretic peptide (BNP) in cardiac tissue samples (Figure S1). M‐mode echocardiography was performed to assess cardiac function. The ejection fraction (EF) was significantly reduced in diabetic mice, indicating an impaired systolic function during diabetes, while Cos treatment rescued these pathological changes (Figure 1G,H, Table S2). Overall, these findings suggest that Cos improves cardiac function in diabetic mice and alleviates hyperglycaemia‐induced cardiac pathological injuries.

### 3.2 Cos attenuates diabetes‐induced cardiac fibrosis and hypertrophy

**FIGURE 2:**
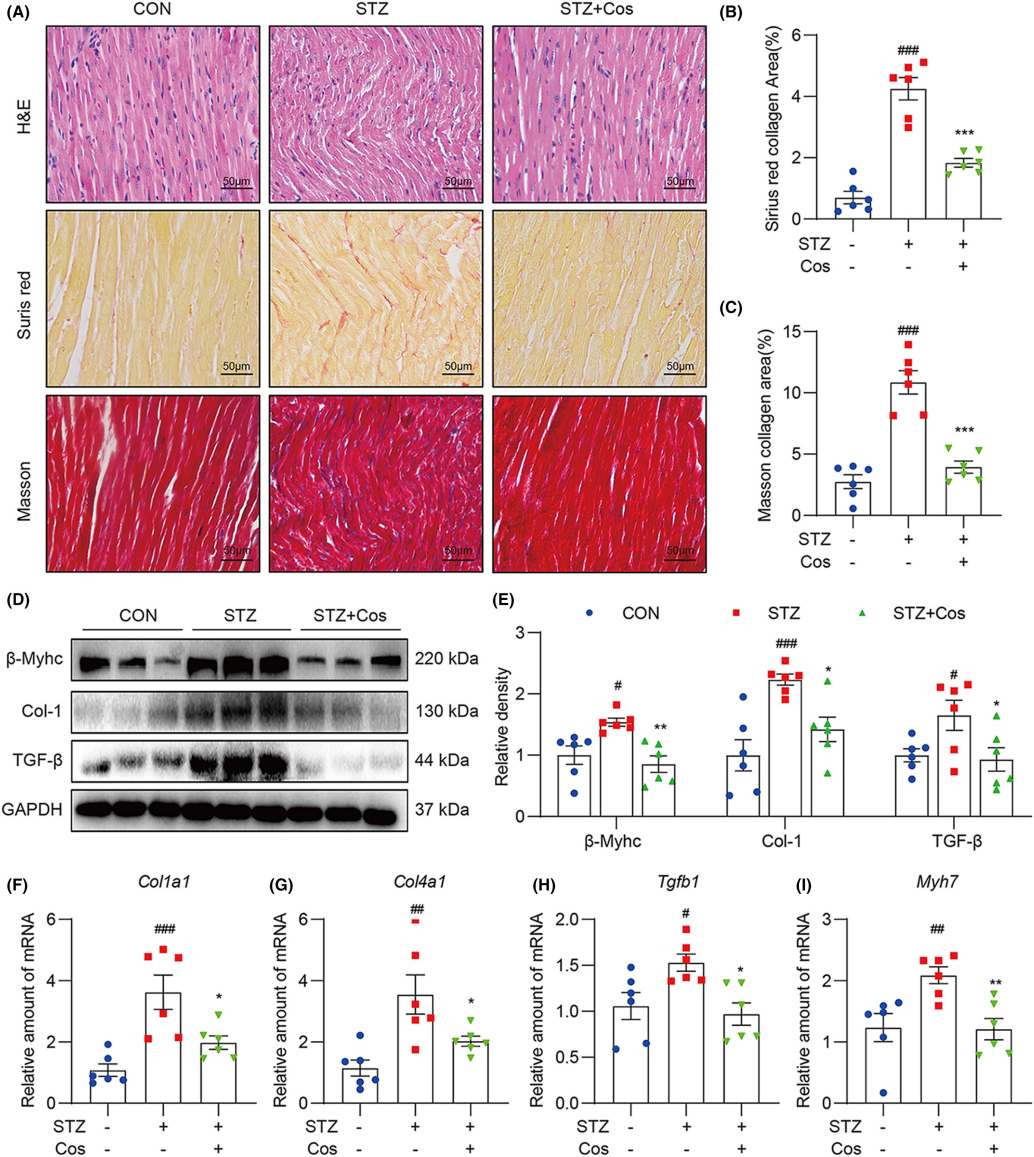
Cos attenuates diabetes‐induced cardiac fibrosis and hypertrophy. (A) H&E, Sirius red and Masson's trichrome staining were performed. (B,C) Quantification of positive collagen area in Sirius red and Masson's trichrome staining results. (D) Western blot analysis was used to detect protein levels of Col‐1, TGF‐β and β‐Myhc in heart tissues. GAPDH was used as a loading control. Relative density was quantified using ImageJ (E). (F–I) Relative mRNA levels of *Col1a1*, *Col4a1*, *Tgfb1* and *Myh7*. Data were normalized to β‐actin and expressed as mean ± SEM, *n* = 6. #*p* < 0.05, ##*p* < 0.01, ###*p* < 0.001 between CON and STZ groups; *p < 0.05, **p < 0.01, ***p < 0.001 between STZ and STZ + Cos groups.

Next, we evaluated structural alternations in the heart tissues of diabetic mice. H&E staining revealed that diabetic hearts exhibited structural abnormalities, including broken fibres, disordered myocardial structures and the presence of foci with necrotic myocytes. Treatment with Cos reversed diabetes‐induced structural alternations in the heart (Figure 2A). Sirius red‐ and Masson's trichrome‐stained sections were used to examine collagen deposition and fibrosis in cardiac tissues. Compared with the heart tissues of the control group, the hearts of diabetic mice showed collagen deposition. However, Cos treatment significantly decreased the upregulation of collagen fibres (Figure 2A–C). Consistent with the staining results, Western blot analysis also demonstrated the Cos‐mediated protective effects against cardiac fibrosis and hypertrophy. The heart tissue samples of Cos‐treated diabetic mice exhibited decreased protein levels of pro‐hypertrophic (β‐Myhc) and profibrotic (type 1 collagen; transforming growth factor β) markers when compared with those in the STZ‐induced diabetic mice (Figure 2D,E). Additionally, Cos treatment downregulated transcription levels of *Col1a1*, *Col4a1*, *Tgfb1* and *Myh7* (Figure 2F–I). These findings indicate that Cos administration attenuates cardiac fibrosis and hypertrophy in DCM mice.

### 3.3 Cos mitigates hyperglycaemia‐mediated myocardial inflammation and oxidative stress

**FIGURE 3:**
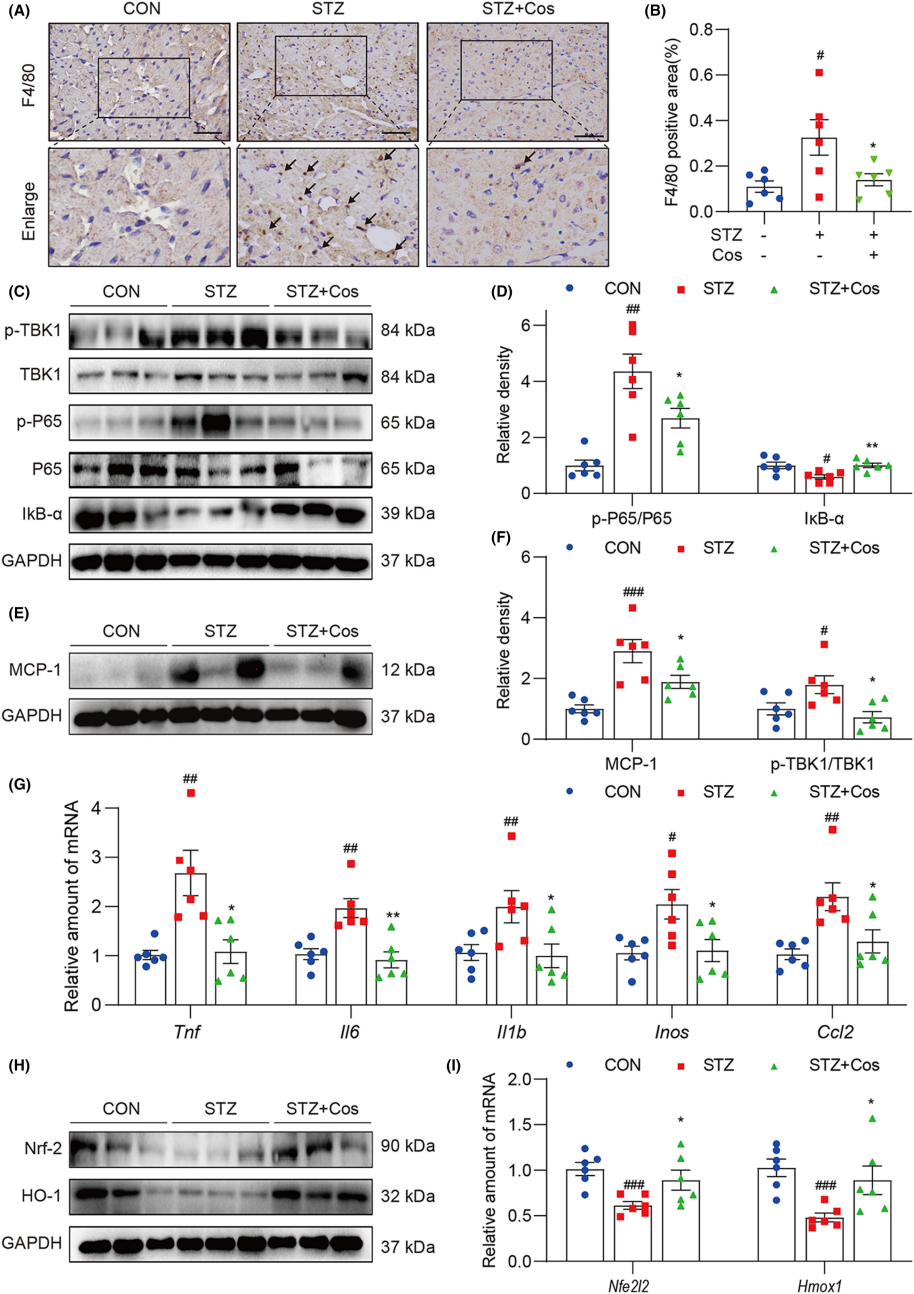
Cos mitigates hyperglycaemia‐induced myocardial inflammation and oxidative stress. (A) Immunohistochemistry for F4/80 was performed to indicate macrophage infiltration into the hearts of diabetic mice. (B) Quantification of F4/80 positive staining intensity. (C) Representative immunoblot for p‐P65, P65, p‐TBK1, TBK1 and IκB‐α in cardiac tissues of mice. Densitometric quantification was performed using ImageJ (D). (E) Western blotting was performed to determine the protein level of MCP‐1. Relative density is shown in (F). (G) Relative mRNA levels of *Tnf*, *Il6*, *Il1b*, *Inos* and *Ccl2* in cardiac tissues. Data were normalized to β‐actin. (H) Protein levels of Nrf‐2 and HO‐1 were determined by Western blotting. (I) Relative levels of Nrf‐2 and HO‐1 mRNA were normalized to β‐actin. Data are expressed as mean ± SEM, *n* = 6. #*p* < 0.05, ##*p* < 0.01, ###*p* < 0.001 between CON and STZ groups; *p < 0.05, **p < 0.01, ***p < 0.001 between STZ and STZ + Cos groups.

Macrophage infiltration and myocardial inflammation are well‐established events in the pathogenesis of DCM.^13^, ^14^ We then performed F4/80 immunohistochemical staining to examine macrophage infiltration in the diabetic heart tissues. As shown in Figure 3A,B, Cos treatment significantly reduced macrophage infiltration in cardiac tissues of diabetic mice. To clarify the influence of Cos on hyperglycaemia‐induced myocardial inflammation, we examined the activation of the NF‐κB signalling pathway. As shown in Figure 3C,D, Cos treatment markedly suppressed hyperglycaemia‐induced IκB‐α degradation and inhibited the phosphorylation of TBK1 and P65, indicating the anti‐inflammatory effect of Cos in diabetic hearts. MCP‐1 is a key chemokine that plays a pivotal role in diabetes‐induced tissue inflammation.^15^ Cos treatment markedly decreased the MCP‐1 protein level in the diabetic hearts when compared with those of the STZ group (Figure 3 E,F). Moreover, Cos treatment reduced transcription levels of inflammatory cytokines (*Tnf*, *Il6* and *Il1b*) and inflammatory‐related genes (*Inos* and *Ccl2*) (Figure 3G). Taken together, Cos mitigates diabetes‐induced cardiac inflammation by inhibiting NF‐κB activation and macrophage infiltration.

Finally, we examined the Cos‐mediated antioxidant activity and determined whether this antioxidant activity partly contributes to the cardioprotective effect in diabetic heart. Nrf‐2 and haem oxygenase (HO‐1) were examined as indicators of oxidative stress. Protein levels of Nrf‐2 and HO‐1 were reduced in the hearts of STZ‐induced diabetic mice, indicating the impaired antioxidation capability in diabetic hearts. However, the protein and mRNA levels of both Nrf‐2 and HO‐1 were rescued by Cos treatment (Figure 3H,I). In addition, increased ROS level has been well established to play a key role in the pathogenesis of DCM.^16^, ^17^ We further determined ROS level in the cardiac tissues. We found that Cos treatment markedly reduced the production of ROS in the diabetic hearts (Figure S2). Taken together, the cardioprotective effects of Cos might be mediated by its anti‐inflammatory and antioxidant effects.

### 3.4 Pretreatment with Cos inhibits HG‐induced fibrotic and hypertrophic responses in H9c2 cells

**FIGURE 4:**
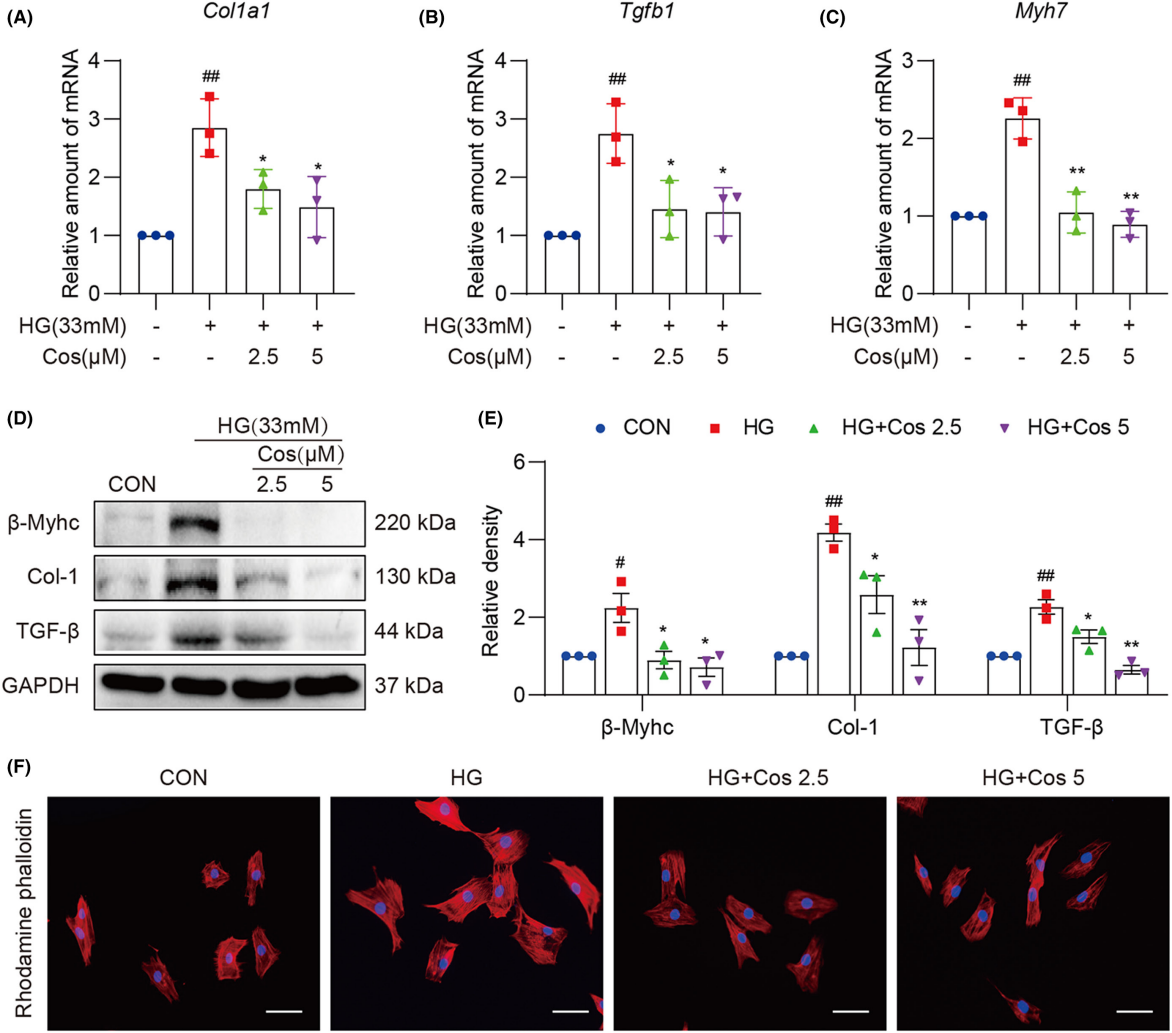
Cos pretreatment inhibits HG‐induced fibrotic and hypertrophic responses in H9c2 cells. (A–C) mRNA levels of *Col1a1*, *Tgfb1* and *Myh7* in HG‐challenged H9c2 cells. Cells were pretreated with Cos for 1 h, followed by exposure of HG for 24 h. (D) H9c2 cells were exposed to HG for 36 h after pretreatment with Cos for 1 h. The protein levels of Col‐1, TGF‐β and β‐Myhc were detected in the cell lysis. The relative density of protein levels was quantified (E). (F) Rhodamine‐phalloidin staining was performed to assess the changes of cell size. H9c2 cells were pretreated with Cos for 1 h, following HG‐treatment for 36 h. Data are expressed as mean ± SEM, *n* = 3. #*p* < 0.05, ##*p* < 0.01, ###*p* < 0.001 between CON and STZ groups; *p < 0.05, **p < 0.01, ***p < 0.001 between STZ and STZ + Cos groups.

H9c2 myocardial cells were selected to evaluate the effect of Cos in vitro. First, optimal Cos dosages were determined using the MTT assay (Figure S3). The dosages of 2.5 and 5 μM of Cos were chosen as appropriate dose for the further in vitro experiments. As shown in Figure 4A–C, HG stimulation for 24 h significantly upregulated mRNA levels of fibrosis‐ (Col‐1 and TGF‐β) and hypertrophy‐related (β‐Myhc) factors, whereas pretreatment with Cos significantly reversed these changes in the HG‐treated H9c2 cells. Likewise, Cos pretreatment reduced protein levels of Col‐1, TGF‐β and β‐Myhc (Figure 4D,E). Furthermore, rhodamine‐phalloidin staining revealed that Cos reduced HG‐induced cellular hypertrophy in H9c2 cells (Figure 4F). These findings indicate that Cos exhibits cardiac protective effect by attenuating HG‐induced fibrotic and hypertrophy responses in myocardial cells.

### 3.5 Cos reduced HG‐induced inflammation and oxidative stress in H9c2 cells

**FIGURE 5:**
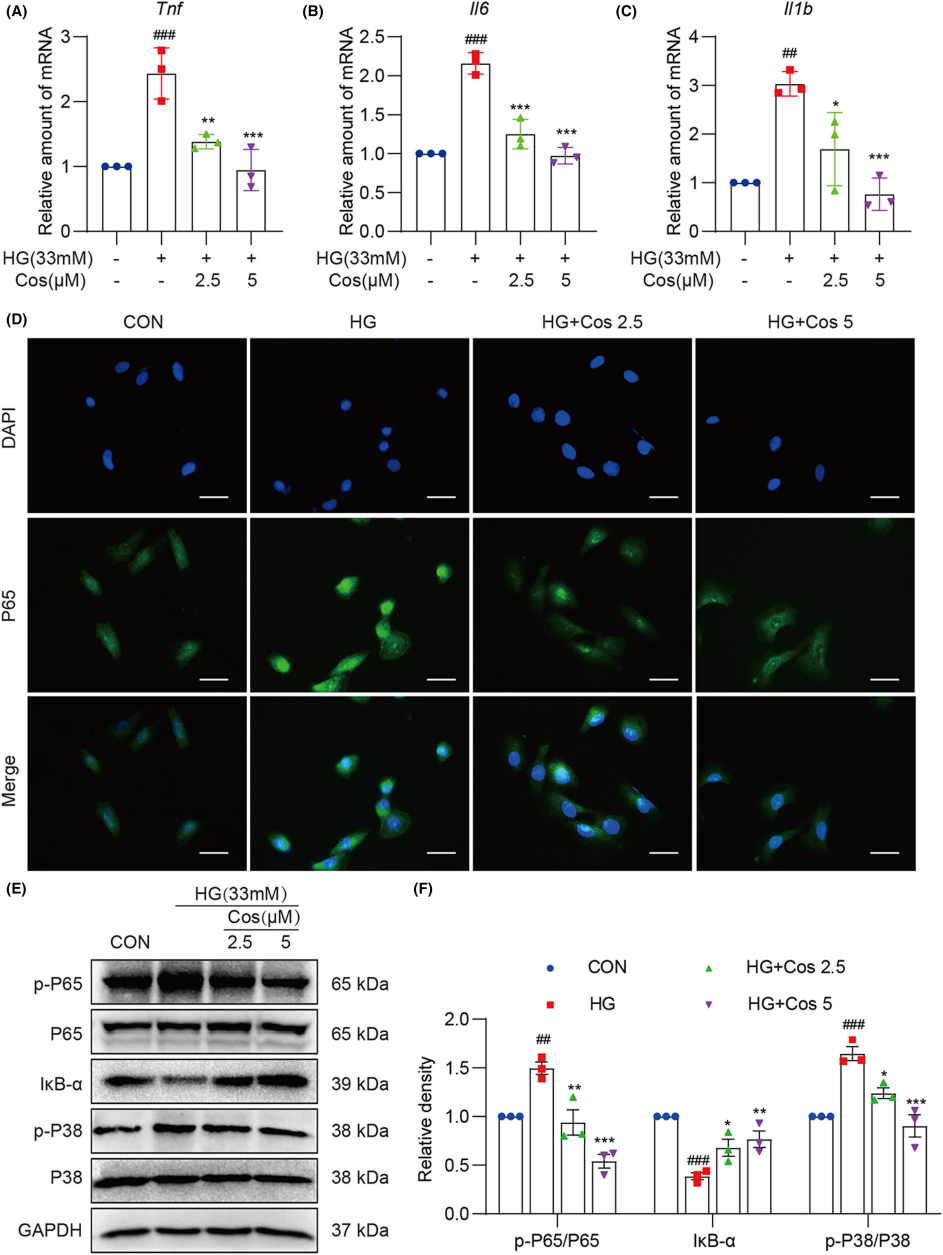
Cos pretreatment reduces HG‐induced H9c2 cell inflammation. (A–C) H9c2 cells pretreated with Cos for 1 h were lysed after a 12‐h exposure to HG. The mRNA levels of *Tnf*, *Il6* and *Il1b* were measured via RT‐qPCR. (D) Immunofluorescence staining of p65 in H9c2 cells. Cells were pretreated with Cos for 1 h and then exposed to HG for 3 h [scale bar = 20 μm]. (E) Protein levels of p‐P65, P65, IκB‐α, p‐P38 and P38 were determined. Cells were pretreated with Cos for 1 h, followed by exposure of HG for 1 h. Densitometric quantification was performed using ImageJ (F). Data are expressed as mean ± SEM, *n* = 3. #*p* < 0.05, ##*p* < 0.01, ###*p* < 0.001 between CON and STZ groups; *p < 0.05, **p < 0.01, ***p < 0.001 between STZ and STZ + Cos groups.

We next investigated the effect of Cos on HG‐induced inflammatory and antioxidative responses in H9c2 cells. As shown in Figure 5A–C, Cos pretreatment reduced HG‐induced upregulation of pro‐inflammatory cytokines such as *Tnf*, *Il6* and *Il1b*. In order to examine NF‐κB activation in HG‐treated H9c2 cells, we performed p65 immunofluorescence staining and assessed NF‐κB activation using Western blotting. As shown in Figure 5D and Figure S4, pretreatment of Cos decreased nuclear p65 levels in HG‐challenged H9c2 cells. Furthermore, we observed that Cos inhibited the phosphorylation of p65 and p38 and reduced IκB‐α degradation (Figure 5E,F). These results suggest that Cos suppresses NF‐κB‐ and p38‐MAPK‐mediated inflammatory cytokine production.

**FIGURE 6:**
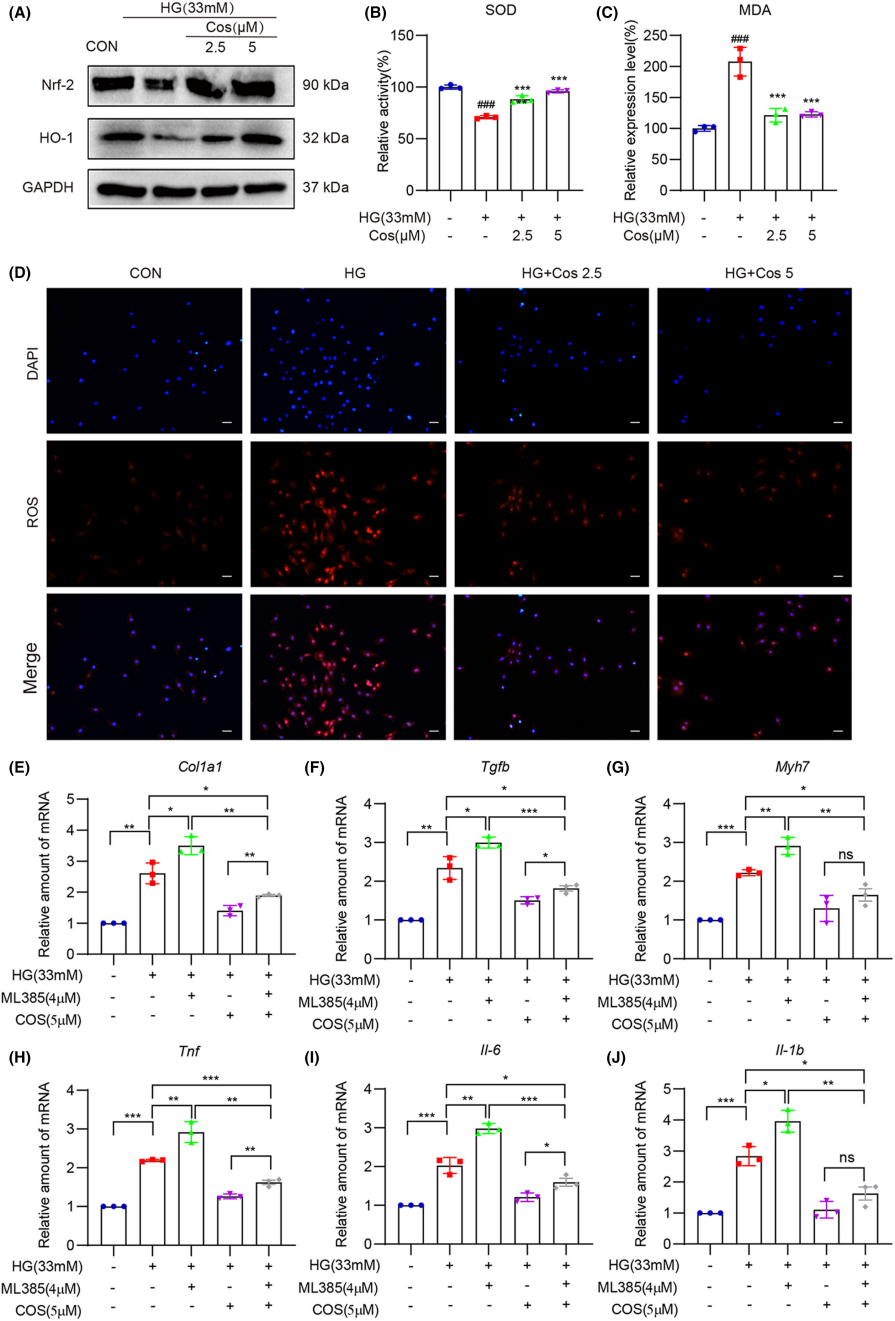
Cos pretreatment reduces HG‐induced oxidative stress in H9c2 cells. (A) Western blot analysis was used to determine the protein levels of Nrf‐2 and HO‐1. GAPDH was used as the loading control. (B–C) Enzymatic activity of SOD and levels of MDA in lysates prepared from H9c2 cells. Cells were exposed to HG for 4 h after pretreatment of Cos for 1 h. (D) Representative images of ROS staining in H9c2 cells. Cells with red fluorescence indicated intracellular ROS. (E–J) H9c2 cells were pretreated with Cos (5 μM) with or without pretreatment of ML385 (4 μM), followed by exposure of HG for 24 h. mRNA levels of *Col1a1*, *Tgfb*, *Myh7*, *Tnf*, *Il6* and *Il1b* were determined. Data were normalized to β‐actin. Data are expressed as mean ± SEM, *n* = 3 per group. *p < 0.05, **p < 0.01, and ***p < 0.001, ns, not significant.

Finally, we confirmed the effect of Cos on HG‐induced oxidative stress in H9c2 cells. As shown in Figure 6A and Figure S5, HG stimulation reduced expression of Nrf‐2 and HO‐1, indicating that HG condition inhibits the Nrf‐2/HO‐1 antioxidation signalling pathway. However, pretreatment with Cos effectively activated the Nrf‐2/HO‐1 signalling pathway in HG‐challenged H9c2 cells. Cos also enhanced the enzyme activity of SOD and decreased the levels of MDA, a natural bi‐product of lipid peroxidation in HG‐treated H9c2 cells (Figure 6B,C). In addition, DHE staining revealed that increased ROS levels caused by HG were significantly reduced by pretreatment of Cos, further confirming the antioxidation of Cos (Figure 6D). Overall, our findings indicate that Cos suppresses HG‐induced inflammation and oxidative stress in H9c2 cells, thereby reducing fibrotic and hypertrophic responses in H9c2 cells.

In order to figure out the underlying links between the anti‐inflammatory and antioxidant effects of Cos, ML385, a Nrf‐2 inhibitor was used in our study. As show in Figure 6E–G, Nrf‐2 inhibition aggravated HG‐induced fibrotic responses and impaired the protective effect of Cos. Meanwhile, the production of inflammatory cytokines was increased by Nrf‐2 inhibition, and the anti‐inflammatory effect of Cos was also weakened by ML385 (Figure 6H–J). However, compared to HG group, the cardioprotective effect and anti‐inflammatory effect of Cos were still exist even Nrf‐2 was inhibited. These data indicate that Cos, at least partly, exhibited its cardiac protection effects via activating Nrf‐2 pathway.

## 4 DISCUSSION

**FIGURE 7:**
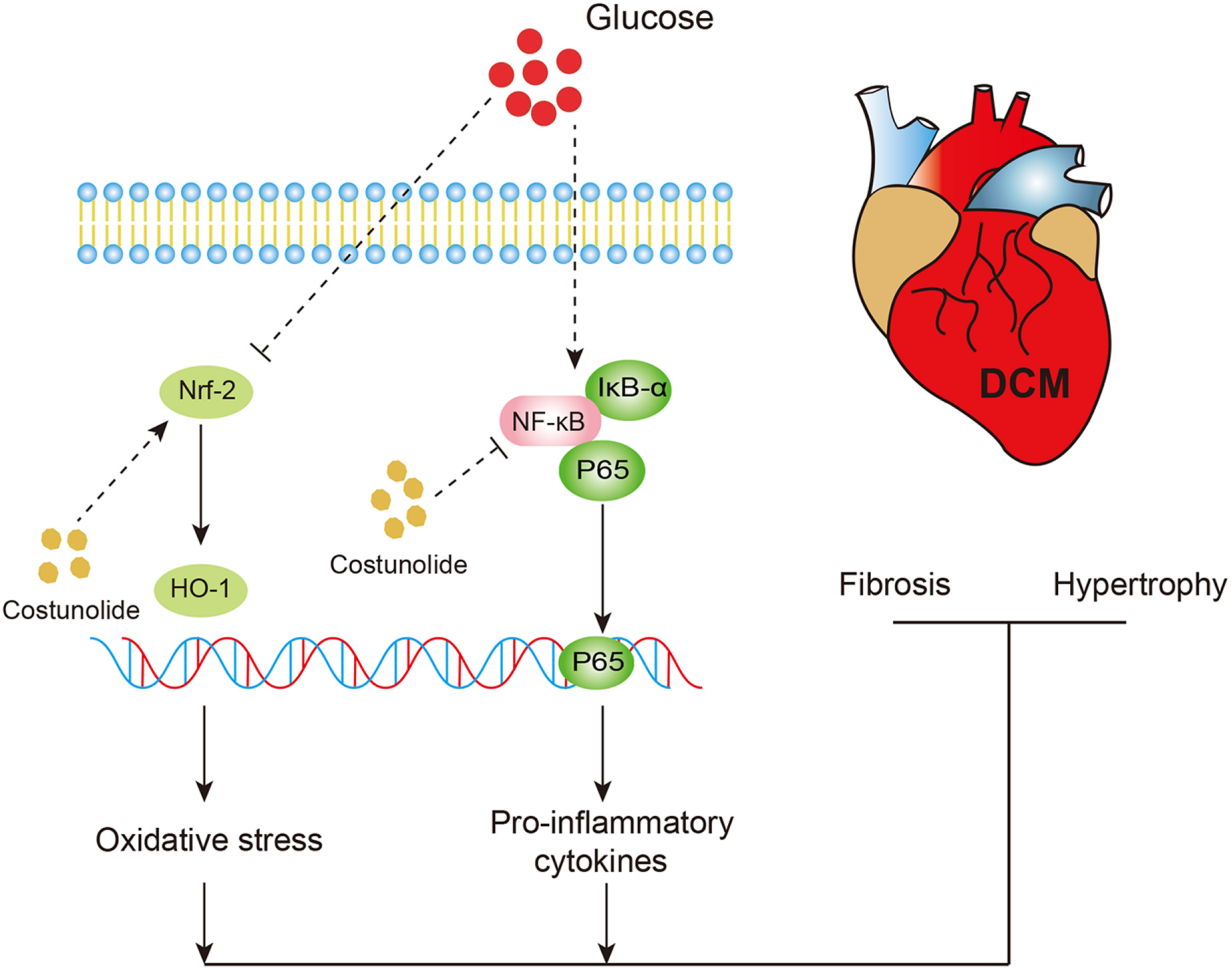
Schematic illustration indicates the potential mechanism of Cos in alleviating hyperglycaemia‐induced diabetic cardiomyopathy.

In recent years, natural products have gained considerable momentum owing to their high efficiency, low toxicity and high accessibility. These properties facilitate the development of natural products into therapeutic drugs for various diseases. Several natural compounds are well‐known to show therapeutic effects against DCM. For example, crocin, mangiferin and kaempferol ameliorate diabetes‐induced cardiac injuries and improve cardiac function in different DCM models.^18^, ^19^, ^20^ These effects are mainly attributed to their anti‐inflammatory and antioxidant properties. Cos is a natural sesquiterpene lactone exhibiting potent anti‐inflammatory activity, demonstrating potential therapeutic utility in diverse diseases such as acute liver injury, osteoarthritis and ulcerative colitis.^8^, ^21^, ^22^ In addition, Cos has been shown to protect against doxorubicin‐induced toxicity in rats by modulating oxidative stress, inflammation and apoptosis.^23^ Thus, the anti‐inflammatory and antioxidative effects of Cos make it a potential candidate for the treatment of DCM. In this study, we show that Cos improved cardiac function and mitigated adverse cardiac remodelling in the STZ‐induced diabetic mouse model. The potential mechanism of Cos in alleviating hyperglycaemia‐induced diabetic cardiomyopathy is shown in Figure 7. Treatment with Cos markedly reversed hyperglycaemia‐associated cardiac fibrosis and hypertrophy. This Cos‐mediated cardioprotective effect was associated with decreased inflammation and oxidative stress via the suppression of NF‐κB‐ and p38‐MAPK‐mediated inflammatory responses, along with the activation of Nrf‐2/HO‐1‐mediated antioxidative responses. Our data indicate that Cos, which has been shown to effectively disrupt both inflammatory response and oxidative stress caused by diabetes, could be a potential therapeutic candidate for the treatment of DCM.

The most prevalent therapeutic strategy for diabetes is treatment of hypoglycaemic drugs with diet and exercise management.^24^ However, hyperglycaemia‐induced tissue injury is inevitable in the progress of diabetes, and there are no clinical drugs for the treatment of diabetic complications.^25^, ^26^, ^27^ Inflammation and oxidative stress are the crucial mechanisms that contribute to hyperglycaemia‐induced cardiac injury.^28^ Blockade of inflammation and oxidative stress can provide potent therapeutic effect in DCM.^17^, ^29^ There are two main advantages of Cos for DCM treatment. Firstly, Eliza et al show that Cos significantly decreased glycosylated haemoglobin (HbA1c) and markedly increased plasma insulin level in diabetic Wistar rats.^30^, ^31^ In their model, the mice were treated orally with Cos every day for 30 or 60 days on the first day the diabetes model was established, indicating that costunolide exhibits hypoglycaemic effect in early stage of diabetes. Secondly, in our study, Cos was orally administered once every 2 days for 8 weeks after T1DM model was established for 8 weeks. Our data show that Cos treatment did not alter body weight and serum glucose levels when compared to the diabetic group, indicating that Cos might not alleviate glycaemic dysfunction if given at the late stage of diabetes. However, Cos treatment inhibited hyperglycaemia‐induced inflammation and oxidative stress, leading to reduced cardiac damage in the end stage of diabetes. All these data prove that Cos can improve DCM during the whole pathology process of diabetes, making Cos as an excellent candidate for the treatment of DCM.

Excessive production of pro‐inflammatory factors and elevated ROS levels have been observed in DCM.^32^ There is an intricate crosstalk between diabetes‐induced inflammatory responses and oxidative stress.^33^, ^34^, ^35^ Recently, Nrf‐2 knockdown was shown to eliminate piceatannol‐ and fortunellin‐mediated anti‐inflammatory effects in DCM.^36^, ^37^ Furthermore, SnPP IX, an HO‐1 inhibitor, reportedly inhibits the anti‐inflammatory function of beta‐naphthoflavone in lipopolysaccharide (LPS)‐induced inflammation in BV‐2 cells.^38^ These findings suggest that the Nrf2/HO‐1 singling pathway may regulate inflammatory responses. Interestingly, oxidative stress may also be influenced by inflammatory responses. TLR4 knockdown was found to weaken NADPH oxidase activity and reduce ROS production in diabetic mice.^39^ Yuan et al. have demonstrated that TAK242, a TLR4 inhibitor, decreases ROS accumulation and inhibits tubular cell apoptosis in diabetic mice.^40^ BAY‐11‐7082, an NF‐κB inhibitor, was shown to upregulate the protein level of Nrf‐2 and reduce ROS production in human osteoarthritic chondrocytes.^41^ These findings suggest that antioxidation may be regulated by TLR4/NF‐κB signalling, thereby providing a new perspective that NF‐κB‐mediated inflammation is closely associated with oxidative stress during the progression of DCM.

The anti‐inflammatory activity of Cos has been extensively reported and attributed to the inhibition of the NF‐κB and MAPK signalling pathways.^22^, ^42^ Cos was also shown to exhibit cardioprotective effect in HFD‐induced obesity mice model^43^ and doxorubicin‐induced toxicity in rats via inhibiting NF‐κB inflammatory signalling pathway.^23^ However, the antioxidant activity of Cos has not been precisely elucidated. An ethanolic extract of *Saussureae Radix* was shown to attenuate neuroinflammation via induction of the Nrf‐2/HO‐1 signalling pathway.^44^ Using high‐performance liquid chromatography analysis, it was confirmed that Cos is one of two major sesquiterpenoids composing the ethanolic extract of *Saussureae Radix*. Therefore, we speculated that Cos exhibits antioxidant activity. Furthermore, Cos is reported to trigger the antioxidative defence system by increasing Nrf‐2 and HO‐1 expression in the LPS‐ and D‐galactosamine‐induced acute liver injury model.^45^ These findings indicate that the antioxidant activity of Cos may be mediated via Nrf‐2 and HO‐1 activation. In the present study, we demonstrated that Cos exhibited cardioprotective effect through inhibiting NF‐κB activation and restoring the suppressed antioxidant Nrf‐2 system. Although we cannot figure out whether the anti‐inflammatory or the antioxidant effect of Cos contributes more important role in treating DCM, we prove that the Nrf2 inhibitor, ML385 significantly reversed the anti‐inflammatory effects of Cos, indicating that Cos, at least partly, exhibited its cardiac protection effects via activating Nrf‐2 pathway.

There are some limitations in the present study. First, we only examined the effect of Cos in a single dosage in vivo. The dosage of Cos at a range of 10–30 mg/kg is frequently used in the treatment of inflammatory diseases. Among them, the dose of 20 mg/kg shows stable anti‐inflammatory activity and strong therapeutic effects in most of the tested models.^22^, ^31^, ^46^ In consideration of the safety and effectiveness, 20 mg/kg was used as the administration dose in our in vivo study. In future investigations, multiple dosages will be examined to establish the pharmacological function of Cos in DCM treatment. In addition, we did not clarify the effect of Cos on cardiomyocyte apoptosis in DCM. It is well‐accepted that myocardial apoptosis is crucial for inducing cardiac fibrosis and hypertrophy. We speculate that the cardioprotective effect of Cos may be partly attributed to its anti‐apoptotic effect. Moreover, studies have shown that Cos suppresses apoptosis of tumour cells in various tumour models.^20^, ^47^, ^48^ Mao et al. have reported the anti‐apoptotic capability of Cos in LPS‐induced acute lung injury,^45^ indicating the anti‐apoptotic potential of Cos in inflammatory diseases. N‐acetyl‐L‐cysteine, an antioxidant and glutathione precursor, can prevent myocardial apoptosis in a type 1 diabetes mouse model.^49^ Therefore, Cos may also suppress myocardial apoptosis in an antioxidative stress manner. However, a comprehensive study is needed to elucidate the possible mechanisms elucidating how Cos regulates both inflammatory and oxidative responses in DCM. Finally, our study demonstrated that Cos inhibited IκB‐α degradation and nuclear translocation of P65. Nevertheless, Cos may inhibit the upstream NF‐κB protein. Cos treatment can reduce TAK1 phosphorylation and inhibit the interaction between TAK1 and TAB1.^42^ In addition, IKK‐β may be another target of Cos, given that Cos inhibits LPS‐induced BV2 microglial inflammation by inhibiting the IKK‐β/NF‐κB signalling pathway.^50^ Parthenolide, a sesquiterpene lactone with a highly similar parent nucleus and double‐bond structure to Cos, can decrease advanced oxidation protein product (AOPP)‐induced MCP‐1 expression by inhibiting the phosphorylation of IKK‐β and NF‐κB p65.^51^ Using molecular docking technology, Mondawood et al. have shown that parthenolide potently binds with IKK‐β. These results indicate that IKK‐β may be the direct target of Cos.^52^ Accordingly, Cos may be a pan‐NF‐κB inhibitor and confer protection against DCM via its anti‐inflammatory activities.

In summary, our study suggests that Cos attenuates cardiac fibrosis and hypertrophy by inhibiting inflammation and oxidative stress in the STZ‐induced mouse model. Furthermore, Cos can reverse HG‐induced H9c2 fibrosis and hypertrophy in vitro. Mechanistically, Cos‐induced anti‐inflammatory and antioxidative effects were mediated by simultaneously inhibiting the NF‐κB signalling pathway and inducing the Nrf2/HO‐1 pathway. Since Cos shows significant hypoglycaemic effect in the early stage of diabetes and provides cardioprotective effect via anti‐inflammatory and antioxidant activities in end stage of diabetes, Cos may be a potential therapeutic candidate for treating DCM.

## AUTHOR CONTRIBUTIONS

**Bo Jin:** Data curation (equal); investigation (lead); methodology (lead). **Yi Chen:** Investigation (equal); methodology (equal); writing – original draft (equal). **Jiong Wang:** Investigation (supporting); validation (supporting); visualization (supporting). **Yue Chen:** Formal analysis (equal); investigation (supporting). **Mengpei Zhang:** Data curation (supporting); investigation (supporting); writing – original draft (supporting). **Jianxiong Huang:** Conceptualization (equal); data curation (equal); project administration (equal); writing – review and editing (equal).

## CONFLICT OF INTEREST STATEMENT

The authors declare no conflict of interest.

## Supporting information


Appendix S1.

## Data Availability

All the data are available from the authors on request.
