# Supplementary material for: Costunolide alleviates hyperglycaemia‐induced diabetic cardiomyopathy via inhibiting inflammatory responses and oxidative stress
Source: J Cell Mol Med. 2023 Feb 21;27(6):831–45. doi: 10.1111/jcmm.17686 (PMC10002915; doi:10.1111/jcmm.17686)
Supplement: Supplementary file 1 — Appendix S1. [file JCMM-27-831-s001.docx]

***Supporting Information***

**Costunolide Alleviates Hyperglycemia-Induced Diabetic Cardiomyopathy by Inhibiting Inflammatory Responses and Oxidative Stress**

**Supplementary Table S1.** Primer sequences for qPCR assay.

| *Gene Name* | *Specie* | *Primers (5` - 3`)* |
| --- | --- | --- |
| *Col1a1* | Mouse | F:TGGCCTTGGAGGAAACTTTG  R:CTTGGAAACCTTGTGGACCAG |
| *Col4a1* | Mouse | F:CTGGCACAAAAGGGACGAG  R:ACGTGGCCGAGAATTTCACC |
| *Tgfb1* | Mouse | F:CCAGATCCTGTCCAAACTAAGG  R:CTCTTTAGCATAGTAGTCCGCT |
| *Myh7* | Mouse | F:CAGAACACCAGCCTCATCAACCAG  R:TTCTCCTCTGCGTTCCTACACTCC |
| *Inos* | Mouse | F:GGAGTGACGGCAAACATGACT  *R:*TCGATGCACAACTGGGTGAAC |
| *Il1b* | Mouse | F:TCGCAGCAGCACATCAACAAGAG  R:AGGTCCACGGGAAAGACACAGG |
| *Il6* | Mouse | F:CTCCCAACAGACCTGTCTATAC  R:CCATTGCACAACTCTTTTCTCA |
| *Tnf* | Mouse | F:ATGTCTCAGCCTCTTCTCATTC  R:GCTTGTCACTCGAATTTTGAGA |
| *Ccl-2* | Mouse | F:TCACCTGCTGCTACTCATTCACCA  R:TACAGCTTCTTTGGGACACCTGCT |
| *Nfe2l2* | Mouse | F:TCTTGGAGTAAGTCGAGAAGTGT  R:GTTGAAACTGAGCGAAAAAGGC |
| *Hmox1* | Mouse | F:GTTGAAACTGAGCGAAAAAGGC  R:GCCGTGTAGATATGGTACAAGGA |
| *Actb* | Mouse | F:CTACCTCATGAAGATCCTGACC  R:CACAGCTTCTCTTTGATGTCAC |
| *Il1b* | Rat | F:CTCACAGCAGCATCTCGACAAGAG  R:TCCACGGGCAAGACATAGGTAGC |
| *Il6* | Rat | F:ACTTCCAGCCAGTTGCCTTCTTG  R:TGGTCTGTTGTGGGTGGTATCCTC |
| *Tnf* | Rat | F:ATGTCTCAGCCTCTTCTCATTC  R:GCTTGTCACTCGAATTTTGAGA |
| *Myh7* | Rat | F:CCAGAACACCAGCCTCATCAACC  R:CACCGCCTCCTCCACCTCTG |
| *Col1a1* | Rat | F:TGTTGGTCCTGCTGGCAAGAATG  R:GTCACCTTGTTCGCCTGTCTCAC |
| *Tgfb1* | Rat | F:GACCGCAACAACGCAATCTATGAC  R:CTGGCACTGCTTCCCGAATGTC |
| *Actb* | Rat | F:AAGTCCCTCACCCTCCCAAAAG  R:AAGCAATGCTGTCACCTTCCC |

**Supplementary Table S2.** Echocardiographic parameters of T1DM mice

| Groups  Parameters | CON | STZ | |
| --- | --- | --- | --- |
|  |  | Vehicle | Cos |
| EF,% | 70.02 ± 7.309 | 57.95 ± 5.624 ## | 68.32 ± 5.077 ** |
| FS,% | 39.16 ± 6.08 | 30.11 ± 3.925 # | 37.16 ± 3.624 ** |
| LVDS（mm） | 2.175 ± 0.309 | 2.545 ± 0.277 | 1.992 ± 0.3733 * |
| LVDD（mm） | 3.567 ± 0.2132 | 3.637 ± 0.3181 | 3.155 ± 0.4651 * |
| LVAW;s（mm） | 1.476 ± 0.121 | 1.719 ± 0.5517 | 1.310 ± 0.3079 |
| LVAW;d（mm） | 1.029 ± 0.1172 | 1.323 ± 0.5419 | 0.919 ± 0.3156 |
| LVPW;s（mm） | 1.310 ± 0.2135 | 0.977 ± 0.2047 | 1.193 ± 0.2892 |
| LVPW;d（mm） | 0.882 ± 0.1484 | 0.730 ± 0.2113 # | 0.798 ± 0.2815 |

Parameters were showed as mean ± SEM, n = 6 per group; # *p* < 0.05, ## *p* < 0.01 and ### *p* < 0.001, CON v.s. STZ group; * *p* < 0.05, ** *p* < 0.01 and *** *p* < 0.001, STZ v.s. STZ+Cos group.


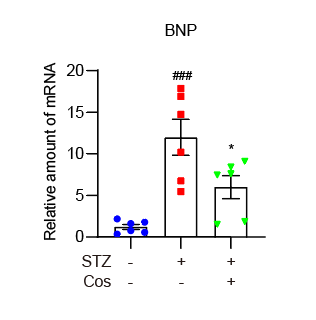


**Supplementary Figure S1.** RT-qPCR analysis of BNP mRNA level in cardiac tissues. Date was normalized to β-actin and were showed as mean±SEM; n = 6 per group. # *p* < 0.05, ## *p* < 0.01 and ### *p* < 0.001, CON v.s. STZ group; * *p* < 0.05, ** *p* < 0.01 and *** *p* < 0.001, STZ v.s. STZ+Cos group.


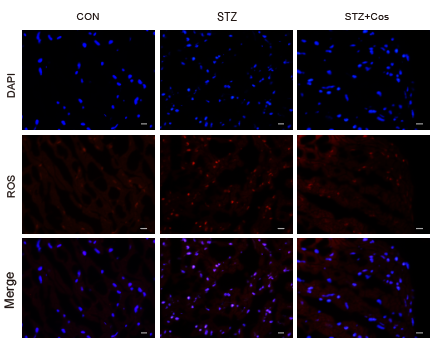


**Supplementary Figure S2.** Intracellular ROS detection via DHE staining in cardiac tissues. Heart tissues with red fluorescence indicated elevated intracellular ROS level [scale bar = 20 μm].


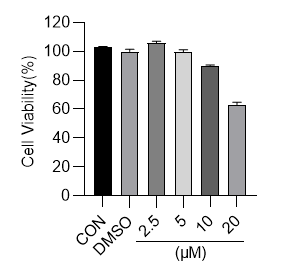


**Supplementary Figure S3.** MTT assay was performed to evaluate the cytotoxicity of Cos in H9c2 cells. The dosage of 2.5 and 5 μM were chosen as appropriate dose for the further *in vitro* experiments.


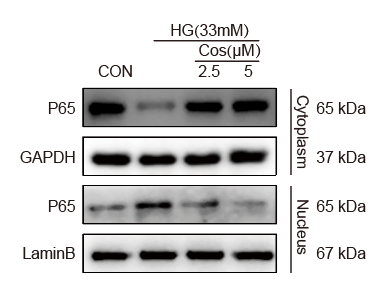


**Supplementary Figure S4.** Immunoblotting analysis of nuclear NF-κB p65 subunit and cytosolic NF-κB p65 levels in H9c2 cells. H9c2 cells were pretreatment with costunolide for 1 h, followed by exposure of HG (33 mM) for 2 h. LaminB and GAPDH were used as loading control for either nuclear and cytosolic proteins.


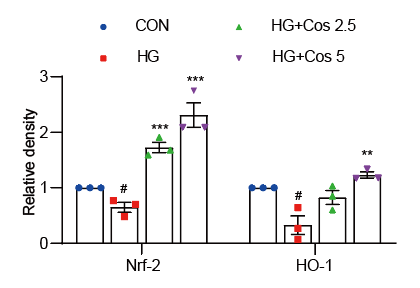


**Supplementary Figure S5.** Relative protein levels of Nrf-2 and HO-1 were quantified by Image J. Data are expressed as mean ± SEM, n=3. # *p* < 0.05, ## *p* < 0.01 and ### *p* < 0.001, CON v.s. HG group; * *p* < 0.05, ** *p* < 0.01 and *** *p* < 0.001, HG v.s. HG+Cos group.
